# Pre-diabetes and cardiovascular risk factors in NAFLD patients: a retrospective comparative analysis

**DOI:** 10.3389/fendo.2025.1416407

**Published:** 2025-02-07

**Authors:** Azam Teimouri, Zahra Ebrahimpour, Awat Feizi, Bijan Iraj, Elahe Saffari, Mojtaba Akbari, Mozhgan Karimifar

**Affiliations:** ^1^ Isfahan Gastroenterology and Hepatology Research Center, Isfahan University of Medical Sciences, Isfahan, Iran; ^2^ Metabolic Liver Disease Research Center, Isfahan University of Medical Sciences, Isfahan, Iran; ^3^ Department of Biostatistics and Epidemiology, School of Public Health, Isfahan University of Medical Sciences, Isfahan, Iran; ^4^ Isfahan Endocrine and Metabolism Research Center, Isfahan University of Medical Sciences, Isfahan, Iran

**Keywords:** non-alcoholic fatty liver disease (NAFLD), pre-diabetes, diabetes, cardiometabolic risk factors, BMI, metabolic syndrome

## Abstract

**Objectives:**

Insulin resistance plays a critical role in the pathophysiology of diabetes mellitus and non-alcoholic fatty liver disease (NAFLD). Moreover, insulin resistance has a central role in atherogensis as the major leading cause of cardiovascular disease (CVD). The aim of the present study was to assess the frequency of pre-diabetes and evaluate the cardiometabolic risk factors among NAFLD patients, comparing those with pre-diabetes to those with normal glucose tolerance.

**Methods:**

In the current retrospective case-control study, the data of 1031 NAFLD patients was retrieved. Based on blood glucose levels, 337 diabetics, 340 pre-diabetes, and, 354 normal glucose patients were diagnosed. After excluding diabetic NAFLD patients, 694 individuals were divided into two groups: normal glucose and pre-diabetes. Various variables, such as age, anthropometric measurements, hypertension, systolic and diastolic blood pressure, and lipid profiles, were extracted from patient files. Statistical analysis was conducted to assess cardiovascular risk factors in NAFLD patients.

**Results:**

Higher age, female gender, higher BMI, triglyceride, waist and hip circumference and waist-to-hip ratio were found in pre-diabetic NAFLD individuals compared with normoglycemic ones (P-value<0.05). Multivariable age-, sex-, BMI- and smoking- adjusted logistic regression showed a predicting role of pre-diabetes and NAFLD concurrence with metabolic syndrome (P-value<0.001, OR:4.31, 95% CI: 2.95- 6.29), but not CVD (P-value=0.353, OR:1.37, 95% CI: 0.71- 2.61).

**Conclusion:**

In this study, nearly one-third of NAFLD patients had pre-diabetes. The mean value of age, BMI, TG, waist and Hip circumference was significantly higher in pre-diabetic patients. The concurrence of pre-diabetes and NAFLD was a predicting factor for metabolic syndrome, but not CVD events.

## Background

Non-alcoholic fatty liver disease (NAFLD) is known as the most common chronic liver disease worldwide (1). Diagnosis of NAFLD is approved when fat saturation consists of more than 5% liver weight in the absence of alcohol overuse and other causes of liver steatosis (2). NAFLD can progress to NASH (non-alcoholic steatohepatitis), advanced fibrosis, cirrhosis, and eventually hepatocellular carcinoma (3). The frequency of NAFLD worldwide is estimated at 25%, and there is an upward trend in the number of NAFLD cases from 83 million in 2015 to 101 million by 2030 in the United States (4). NAFLD mostly occurs in the Middle East and South America (5). The estimated frequency of NAFLD in Iran is nearly 33.9% and it is announced by 39.3% of Isfahan’s population (6–8).

In addition, the population involvement in NAFLD is increasing worldwide due to the obesity epidemic (9). NAFLD can cause primary insulin resistance or may be the result of insulin resistance thus it is strongly associated with components of the metabolic syndrome including obesity, insulin resistance, dyslipidemia, and hypertension (10).

Furthermore, pre-diabetic individuals are up to two folds at increased risk of developing diabetes mellitus. Insulin resistance is the mainstay in the pathophysiology of diabetes mellitus (11–13) which is common between NAFLD and diabetes mellitus (14).

Atherosclerosis is an ongoing inflammatory process leading to the formation of plaques in arterial wall. The pathophysiology of atherogenesis is multifactorial; nevertheless, all the process occurs due to an interaction of pro-inflammation, endothelial bed dysfunction and oxidative stress (15, 16). Surfing the literature has shown a direct and critical association between insulin resistance and atherogenesis. Given that, insulin resistance is associated with increased serum level of pro-inflammatory factors such as interleukin 1 (IL-1), IL-6 and tumor necrosis factor alpha (TNF-α), as well as free oxygen radicals responsible for oxidative stress. Moreover, inappropriate platelet aggregation and calcium plaque deposition in intima-media layer of arteries leading to endothelial dysfunction is well-elucidated in individuals with insulin resistance (17).

Theoretically, as the trace of insulin resistance can be detected in NAFLD and prediabetes, the coincidence of both might make individuals more and more prone to CVDs. A recent meta-analysis of 34,000 patients with NAFLD and insulin resistance over seven years found that NAFLD and insulin resistance increased the risk of mortality and morbidity in CVD by 65%. In fact, the most common cause of death in patients with NAFLD is cardiovascular causes (18). Nevertheless, limited number of researchers have investigated the impact of NAFLD and prediabetes on the incidence of CVD, an issue that is aimed to be evaluated in the current retrospective case-control study.

## Methods study design and participants

**Figure 1:**
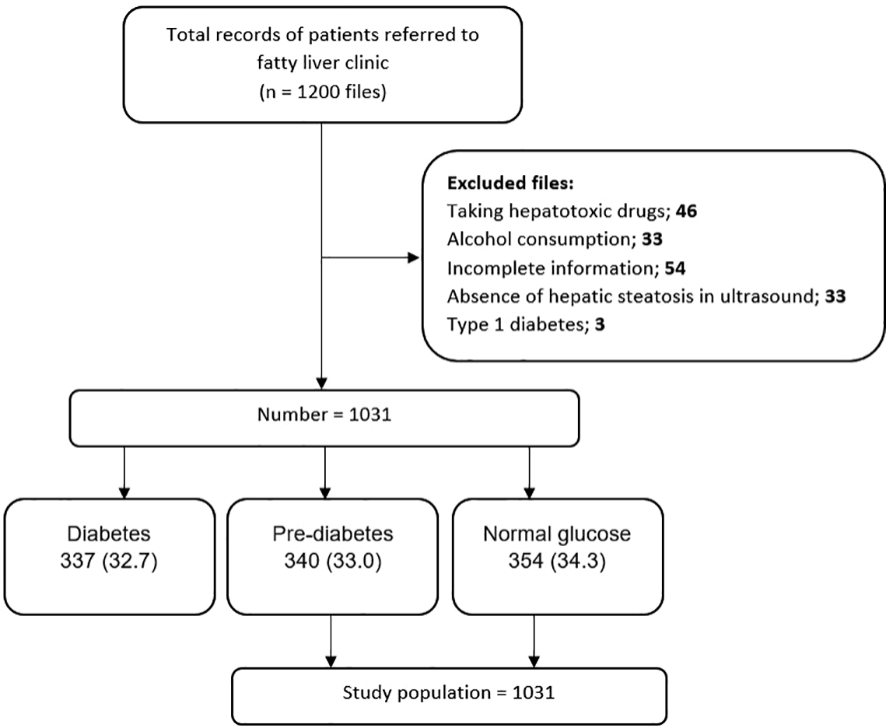
Flow diagram of patient selection process and study design.

This research is a retrospective case-control study. The data of 1200 patients referred to the Fatty Liver Clinic of the Endocrine and Metabolism Research Center, affiliated with Isfahan University of Medical Sciences, from 2012 to 2017 were retrieved. Diagnosis of fatty liver was made based on the presence of hepatic steatosis in the ultrasound available in the files for patients who did not have a history of consuming too much alcohol and was ruled out for other hepatic steatosis reasons. These excluded cases contain chronic liver diseases (e.g., viral and autoimmune diseases) and chronic use of drugs such as glucocorticoids, sodium valproate, methotrexate, amiodarone, tamoxifen, and anti-retroviral agents for HIV. See **Figure 1** for an overview of the statistics. Our data was obtained by considering the history of the diseases and performing some tests, such as checking for viral hepatic markers. The information deficiency in patients’ profiles, especially concerning the main variable of the study, namely the uncertainty of the pre-diabetes status, was not reviewed. Inclusion criteria consisted of NAFLD patients while the exclusion ones were diabetic patients.

### Procedures and assessment of variables

In this study, among the 1,200 considered cases, 1031 NAFLD patients were included, which were subdivided into three groups: group1 = normal glucose, group2 = pre-diabetes, and group3 = type2 diabetes. According to the American Diabetes Association guidelines, the normal glucose criterion is fasting blood glucose (FBS) < 100 mg/dl, the HbA1c < 5.7%, or the blood glucose two hours after using 75 g of glucose (BS 2hr OGTT) < 140 mg/dl. The pre-diabetic criterion is FBS (100 -125 mg/dl), HbA1c (5.7% -6.4%), or BS 2 hr OGTT between 140 and 199 mg/dl. The type2 diabetes criterion is FBS ≥ 126 mg/dl, HbA1c ≥ 6.5%, BS 2hr OGTT ≥ 200 mg/dl, or random plasma glucose ≥ 200 mg/dL in patients with hyperglycemic symptoms (19). The last group, containing 337 type 2 diabetic cases, was excluded after matching the whole 1,031 NAFLD patients to their related groups. Finally, 694 NAFLD patients, groups 1 and 2, were included in the study (**Figure 1**). We evaluated the frequency of pre-diabetes and normal glucose patients and the comparison of cardiovascular risk factors between the prior mentioned groups in NAFLD patients.

Information such as age, gender, height, weight, waist, hip and wrist circumference, medical and drug history, the status of pre-diabetes and diabetes, hypertension, systolic and diastolic blood pressure, lipid profiles (Total Cholesterol, LDL Cholesterol, HDL Cholesterol, and Triglyceride (TG)), Serum Glutamic Oxaloacetic Transaminase (SGOT), Serum Glutamic Pyruvic Transaminase (SGPT), and degree of the fatty liver based on ultrasound were extracted from the files. All the measures were done at the time of NAFLD diagnosis when the patients were primarily diagnosed as NAFLD and referred to our clinic for the first time.

We considered the usage of antihypertensive drugs, a patient’s statement of blood pressure, registered systolic blood pressure of ≥140 (mm Hg), or diastole blood pressure of ≥ 90 (mm Hg) as hypertension or a sign of high blood pressure (20). BMI was calculated by dividing weight (kg) by height squared (m^2^). We categorized this index into 6 groups: BMI<18.5 as less than normal, 18.5-24.9 as normal, 25-29.9 as overweight, 30-34.99 as obesity grade one, 35-39.9 as grade two obesity, and ≥40 as grade three obesity. Waist-hip circumference ratio (WHR) was considered abnormal for values more than 0.9 in women and more than 1 in men. According to the Adult Treatment Panel III, defining the metabolic syndrome, the presence of three or more of the following characteristics was considered as the presence of metabolic syndrome: Waist circumference > 102 cm in men and > 88 cm in women, triglyceride levels ≥ 150 mg/dl, HDL levels < 40 mg/dl in men and < 50 mg/dl in women, systolic blood pressure ≥ 130 mmHg or diastolic blood pressure ≥ 85 mmHg, fasting plasma glucose levels ≥ 100 mg/dl, or drug treatment for elevated blood glucose. For SGPT and SGOT enzymes, we considered values ≤ 40 U/L as normal, 41-120 U/L as more than normal, and values> 120 U/L as much more than normal (20). The stages of NAFLD were determined using ultrasound data in the patient records. Those with at least two ultrasounds, with an equal degree of hepatic contrast, were classified into one of these three categories: mild steatosis, moderate steatosis, and severe steatosis. We recorded any patient who had a history of myocardial infarction, unstable angina, angina pectoris, coronary artery stenosis or any type of revascularization intervention, heart failure, or hospitalization due to heart disease as cardiovascular disease (CVD). The patients with CVD history was requested to bring their medical records in order to confirm the diagnosis. These assessments were done at the time of NAFLD diagnosis when the patients referred to our clinic for the first time.

The Ethics Committee of Isfahan University of Medical Sciences approved the current study (code: IR.MUI.MED.REC.1399.326).

### Statistical analysis

Continuous and categorical variables are reported as mean ± standard deviation (SD) and frequency (percentage). The normality of continuous variables was evaluated using the Kolmogorov-Smirnov test and Q-Q plot, and non-normally positive skewed distributed data such as lipid indices were subjected to logarithmic transformation. Continuous and categorical data were compared between pre-diabetics and normal glucose NAFLD patients using independent samples t-test (or non-parametric Mann-Whitney U test) and chi-square tests. Multiple binary logistic regressions in crude and adjusted models were used to evaluate the risk of (odds ratio) hypertension, cardiovascular disease, and metabolic syndrome in pre-diabetic NAFLD patients compared to normal blood glucose ones. The data were controlled for age, sex, BMI and smoking in the adjusted model. All statistical analyses were done using the Statistical Package for Social Sciences (SPSS, version 16.0 for Windows, 2006, SPSS, Inc, Chicago, IL). P-value < 0.05 was considered statistically significant.

## Results

In this study, 1031 patients with NAFLD were included, of which 354 (34.3%) had normal glucose, 340 (33%) were pre-diabetic, and 337 (32.7%) had type 2 diabetes (**Figure 1**).

After excluding patients with type 2 diabetes, a total of 694 individuals with NAFLD (51% with normal glucose and 49% with pre-diabetes) with a mean age of 45.94 ± 11.26 were included in the analysis.

Out of the 354 people with normal glucose and 340 people with pre-diabetes, 43.9% and 56.1% were female, respectively.

**Table 1** presents the frequency of pre-diabetes across categories of age, gender, and anthropometric indices groups. The frequency of pre-diabetes in older people and females was significantly higher (P< 0.0001). In addition, we observed an increasing trend in terms of pre-diabetics along with increasing BMI, from 0 for BMI< 18.5 kg/m^2^ to 63% for 35 ≤BMI < 39.9 kg/m^2^ (P<0.0001). The frequency of pre-diabetes in NAFLD patients with abnormal WHR was significantly higher than in people with normal WHR (P<0.0001).

**Table 1:** Demographic and anthropometrics characteristics of study population by group.

Characteristics		Group		p-value
		Normal glucose (n=354)	Pre-diabetes (n=340)	
Age (year)	≤ 30	56 (80.0)	14 (20.0)	< 0.0001
	31- 40	96 (62.3)	58 (37.7)	
	41 - 50	100 (51.3)	95 (48.7)	
	51 - 60	73 (36.1)	129 (63.9)	
	> 60	27 (39.7)	41 (60.3)	
Sex (Male/Female)		184/170	123/217	< 0.0001
BMI (kg/m²)	< 18.5	0	0	< 0.0001
	18.5 - 24.9	33 (56.9)	25 (43.1)	
	25 - 29.9	154 (56.6)	118 (43.4)	
	30 - 34.9	120 (49.0)	125 (51.0)	
	35 - 39.9	32 (36.4)	56 (63.6)	
	≥ 40	9 (40.9)	13 (59.1)	
Abnormal WHR^*^		88 (36.8)	151 (63.2)	< 0.0001

Data are presented as Number and Number (Percent).

P-values calculated by chi-square test.

*WHR >1 for males and >0.9 for females.

BMI, Body mass index; WHR, waist–hip ratio.

The frequency of pre-diabetes was not significantly different across SGPT and SGOT and NAFLD grades (P>0.1) (**Table 2**).

**Table 2:** The liver enzymes and fatty liver grade in study population by group.

Characteristics		Group		p-value
		Normal glucose (n=354)	Pre-diabetes (n=340)	
SGPT (U/L)	≤ 40	168 (52.2)	154 (47.8)	0.967
	41-120	176 (51.5)	166 (48.5)	
	> 120	6 (54.5)	5 (45.5)	
SGOT (U/L)	≤ 40	278 (54.5)	232 (45.5)	0.603
	41-120	65 (49.6)	66 (50.4)	
	> 120	1 (50.0)	1 (50.0)	
^*^ FLD Grade	I	163 (55.8)	129 (44.2)	0.147
	II	156 (46.3)	181 (53.7)	
	III	35 (53.8)	30 (46.2)	

Data are presented as Number (Percent).

P-values calculated by chi-square test.

*FLD was determined based on ultrasound findings.

SGPT, Serum Glutamic Pyruvic Transaminase; SGOT, Serum Glutamic Oxaloacetic Transaminase; FLD, Fatty liver disease.

**Table 3** presents the mean value of different cardiometabolic variables in pre-diabetic and normal glucose NAFLD patients. Among them, the mean value of BMI (P<0.0001), TG (p<0.0001), waist (P<0.0001), hip circumference (P=0.014), and WHR (p=0.008) were significantly higher in pre-diabetic than normal glucose cases.

**Table 3:** Comparison of anthropometric and cardiometabolic characteristics in study population by group.

Characteristics	Group		p-value
	Normal glucose (n=354)	Pre-diabetes (n=340)	
BMI (kg/m²)	29.9 ± 4.3	31.1 ± 4.5	< 0.0001
SBP (mmHg)	119.6 ± 13.6	120.5 ± 14.3	0.598
DBP (mmHg)	80.0 ± 9.3	79.1 ± 9.1	0.397
TG (mg/dl)	176.5 ± 89.6	195.8 ± 117.1	0.017
T chol (mg/dl)	196.0 ± 39.4	199.2 ± 39.5	0.295
HDL (mg/dl)	44.1 ± 10.6	43.7 ± 10.2	0.652
LDL (mg/dl)	115.0 ± 32.7	116.6 ± 33.8	0.554
SGPT (U/L)	44.9 ± 28.4	41.1 ± 25.0	0.071
SGOT (U/L)	31.7 ± 17.8	30.2 ± 14.6	0.241
WC (cm)	97.6 ± 10.6	100.4 ± 10.7	< 0.0001
HC (cm)	105.5 ± 7.9	107.1 ± 8.5	0.014
WrC (cm)	17.1 ± 1.3	17.1 ± 1.2	0.866
WHR	0.92 ± 0.08	0.94 ± 0.08	0.008

Data are presented as Mean ± 1 SD.

P-values calculated by Independent sample T- test.

BMI, Body mass index; SBP, Systolic blood pressure; DBP, Diastolic blood pressure; TG, Triglycerides; T Chol, Total cholesterol; HDL, High Density Lipoprotein cholesterol; LDL, Low Density Lipoprotein cholesterol; SGPT, Serum Glutamic Pyruvic Transaminase; SGOT, Serum Glutamic Oxaloacetic Transaminase; WHR, waist–hip ratio; SGPT, Serum Glutamic Pyruvic Transaminase; SGOT, Serum Glutamic Oxaloacetic Transaminase; FLD, Fatty liver disease; WC, waist circumference; HC, Hip circumference; WrC, Wrist circumference.

The comparison of various parameters in terms of fatty liver disease grading but regardless of the patients’ glycemic status revealed statistically that BMI (P-value<0.001), serum triglyceride (P-value=0.001), SGPT (P-value=0.026), waist circumference (P-value<0.001), hip circumference (P-value<0.001) and wrist circumference (P-value<0.001) statistically differed in individuals with different severity of fatty liver disease. Detailed data are demonstrated in **Table 4**.

**Table 4:** Comparison of anthropometric and cardiometabolic characteristics in study population based on FLD grading.

Characteristics	FLD grade			p-value
	I (n=261)	II (n=295)	III (n=57)	
BMI (kg/m²)	29.34 ± 4.37	31.06 ± 4.23	32.03 ± 4.35	< 0.001
SBP (mmHg)	119.38 ± 14.11	121.72 ± 13.44	121.00 ± 8.90	0.442
DBP (mmHg)	79.75 ± 9.02	79.59 ± 9.04	81.00 ± 5.41	0.848
TG (mg/dl)	169.53 ± 101.47	192.69 ± 107.20	222.16 ± 116.49	0.001
T chol (mg/dl)	194.16 ± 36.16	200.82 ± 39.74	197.32 ± 44.32	0.139
HDL (mg/dl)	43.96 ± 11.07	43.97 ± 9.99	42.64 ± 10.29	0.673
LDL (mg/dl)	113.14 ± 32.12	117.52 ± 33.01	116.74 ± 38.87	0.322
SGPT (U/L)	41.13 ± 23.97	45.25 ± 29.83	51.02 ± 25.54	0.026
SGOT (U/L)	29.69 ± 13.99	32.10 ± 18.70	34.26 ± 14.75	0.097
WC (cm)	96.12 ± 11.05	100.71 ± 9.67	102.29 ± 11.42	< 0.001
HC (cm)	104.69 ± 8.49	106.64 ± 7.37	109.38 ± 8.52	< 0.001
WrC (cm)	16.85 ± 1.22	17.38 ± 1.25	17.64 ± 1.32	< 0.001

Data are presented as Mean ± 1 SD.

P-values calculated by One-way ANOVA.

BMI, Body mass index; SBP, Systolic blood pressure; DBP, Diastolic blood pressure; TG, Triglycerides; T Chol, Total cholesterol; HDL, High Density Lipoprotein cholesterol; LDL, Low Density Lipoprotein cholesterol; SGPT, Serum Glutamic Pyruvic Transaminase; SGOT, Serum Glutamic Oxaloacetic Transaminase; WHR, waist–hip ratio; SGPT, Serum Glutamic Pyruvic Transaminase; SGOT, Serum Glutamic Oxaloacetic Transaminase; FLD, Fatty liver disease; WC, waist circumference; HC, Hip circumference; WrC, Wrist circumference.

We conducted a comparative analysis to examine the frequency of CVD, hypertension, and metabolic syndrome in patients diagnosed with NAFLD, specifically comparing those with pre-diabetes to individuals with normal glucose levels (**Table 5**). Our findings revealed a significantly higher frequency of metabolic syndrome (P<0.0001) and history of CAV (P=0.03) in patients with pre-diabetes. However, no significant association was observed between blood glucose levels and hypertension in two groups (P > 0.05). Further logistic regression investigations showed that pre-diabetic status was a standalone predictor of metabolic syndrome in both unadjusted and adjusted models. Given that, the NAFLD individuals with pre-diabetes was at 4.62 (P-value<0.001, OR:4.62, 95% CI: 3.25- 6.55) and 4.31 (P-value<0.001, OR:4.31, 95% CI: 2.95- 6.29) times increased risk of developing metabolic syndrome in unadjusted and adjusted logistic regression analyses, respectively. Furthermore, CVD was 2 folds (P-value=0.032, OR:2 95% CI: 1.06- 3.76) more among pre-diabetic NAFLD patients compared with normoglycemic ones in unadjusted model, while the adjustments for age, sex, BMI and smoking led to an insignificant role for CVD (P-value=0.353, OR:1.37, 95% CI: 0.71- 2.61).

**Table 5:** The frequency of cardiovascular risk factors in patients with NAFLD by group.

Characteristics	Group		p-value	OR (95% CI)		P-value (unadjusted)	P-value (adjusted)
	Normal glucose (n=354)	Pre-diabetes (n=340)		Unadjusted	Adjusted *		
HTN	85 (24)	96 (28.2)	0.205	1.28 (0.88-1.88)	0.76 (0.49-1.17)	0.196	0.219
MetS	86 (24.4)	198 (58.2)	< 0.001	4.62 (3.25-6.55)	4.31 (2.95-6.29)	<0.001	<0.001
CVD	16 (5.1)	29 (9.7)	0.03	2.00 (1.06-3.76)	1.37 (0.71-2.61)	0.032	0.353

Data are presented as Number (Percent).

OR and P-values calculated by logistic regression and * adjustment was made for Age, Sex, BMI and Smoking.

NAFLD, Non-alcoholic fatty liver disease; OR, Odda ratio; CI, Confidence interval; HTN, Hypertension; MetS, Metabolic Syndrome; CVD, Cardiovascular disease.

## Discussion

The main scope of the current study was to investigate the cardiometabolic parameters among the NAFLD individuals with pre-diabetes. Accordingly, we found that those NAFLD meeting pre-diabetes criteria were generally older and predominantly consisted of female gender. Except for triglyceride, other cardiometabolic parameters did not differ with normoglycemic individuals; however, anthropometric indices including BMI, WC, HC and WHR were remarkably higher among pre-diabetic cases. Further evaluations revealed that concurrent NAFLD and pre-diabetes was accompanied by more than 4 times increased risk of metabolic syndrome development, but we found no role for concurrence of NAFLD and prediabetes to predict hypertension or CVD.

In our study, the considered NAFLD population with including criteria, the frequency of pre-diabetes patients is 49%. This percentage is similar to a study conducted by Said Taharboucht et al. in Algeria in 2020 on 213 NAFLD patients among whom prediabetes was detected in 85 individuals accounting for 40% (21). Nevertheless this rate variably differs in other studies. For instance, Cuthbertson and colleagues perform an ongoing investigation of NAFLD in Finland finding that 25.44% of their cases developed prediabetes (22). Another study by Kitazawa et al. reported that 2.96% (770/26,014 cases) of individuals with NAFLD met the criteria for prediabetes, while 26.1% (777/2977 cases) were high-risk individuals for prediabetes development (23). This rate is while the only other study conducted in this field in Iran by Pakzad et al. on 80 NAFLD patients reported a 55% frequency of pre-diabetes (24). Logically, this considerable variability can be attributed to several factors such as the differences in the definition of NAFLD and prediabetes, the sample population, the study design and the determined criteria for including the individuals

We observed that pre-diabetic NAFLD individuals were generally older than normoglycemic ones. Similarly Song et al. presented that NAFLD cases with abnormal glycemic state compatible with prediabetes are older (25). Cuthbertson and colleagues also stated a similar pattern of age distribution among NAFLD individuals developing pre-diabetes (22).

The number of pre-diabetic women was significantly higher than that of pre-diabetic men. This finding is consistent with what was presented by Kitazawa et al. who presented that females are more prone to develop pre-diabetes than men (23). This outcome has been confirmed in other studies, as well (26–28). It seems that the lipid distribution and metabolism as well as hormone differences might be responsible for higher risk of diabetic changes in women suffering NAFLD (26).

We found a significant association between increased BMI, waist circumference, hip circumference, waist-to-hip ration and abnormal WHR and pre-diabetes in NAFLD. These measures have been widely experimented in the literature with firm verifying documentations (26, 29–31). It is clear that obesity has a direct association with NAFLD. On the other hand, it is well-elucidated that obese people are at increased risk of developing diabetic changes as adipose tissue releases increased amounts of non-esterified fatty acids, glycerol, hormones, proinflammatory cytokines and other factors that are involved in the development of insulin resistance (32).

Since long time ago BMI was an indicator of obesity; however, nowadays we know that BMI has significant limitation to distinguish excess adipose tissue. Accordingly, other parameters such as waist and hip circumference and their ration as the indices of peripheral adipose tissue have been emerged revealing a better association with body metabolism, insulin resistance and diabetes development (33–35). Nevertheless, our findings regarding higher measures of these indices in prediabetic NAFLD individuals compared with normoglycemic ones are in agreement with the evidence.

In the current study, we found no significant relationship between SGOT and SGPT enzyme values as well as the grading of fatty liver disease in pre-diabetes compared to normal glucose. The same result was posed in a study by Komshilova et al.; they believed that normal liver enzymes did not rule out necrosis, inflammation, and fibrotic changes in the liver, and up to 70% of patients with NAFLD may have normal enzymes (36). Taharboucht et al., represented a similar observation (21).

The current study showed a clear relationship between increased triglyceride in pre-diabetic NAFLD patients compared to another group. This result is in line with the Indian study (30) and the one in Algeria (21). However, in the present study, there was no significant relationship between cholesterol, LDL, and HDL in pre-diabetes compared to normal glucose patients. Although, it should be mentioned that some of our patients used statins, which lead to lower cholesterol and LDL (37). Therefore, the findings regarding cholesterol-related lipid profile can be considered as source of bias that needs further evaluations.

The logistic regression evaluations revealed that pre-diabetes in NAFLD was a predictor of metabolic syndrome, but not CVD. The predicting role for metabolic syndrome seems logical considering the associations presented above, however we assume that the small number of study population with CVD is responsible for this lacking of association. Moreover, the observational design of our study limits its ability to generalize the prognostic role of pre-diabetes in NAFLD for CVD events.

It is well-evidenced that a large number of NAFLD individuals are prone to develop impaired glucose tolerance or impaired fasting glucose, pre-diabetes and progressively, come down with diabetes mellitus (31, 38–42). This fact majorly contributes to the similar pathogenesis of NAFLD and pre-diabetes, insulin resistance. Given that, insulin resistance is associated with the release of a myriad of lipid metabolites, proinflammatory cytokines and hepatokines (e.g., fetuin A, fetuin B and angiopoietin‐like protein) which in turn leads to developing diabetes mellitus (43, 44). This pathogenesis is similar in pre-diabetic patients, as well; whereas, it is confirmed that up to 70% of them would turn into diabetes mellitus (45).

Moreover, the nature of NAFLD is associated with increased risk of CVD independent of the presence of hypertension, diabetes, and obesity by endothelial dysfunction (46). This relation can be attributed to the pathogenesis of insulin resistance in NAFLD contributing in the process of atherogensis through oxidative stress, endothelial dysfunction and excessive proinflammation (15, 16, 47, 48).

Although in line with our findings, Hubbard et al. presented no heightened risk for CVD in patients with pre-diabetes (49); theoretically, the individuals with pre-diabetes with a similar pathogenesis are at increased risk of developing CVD (50).

Despite the strong points of our study among which the large study population can be named, this investigation meets several limitations. One of the limitations of this study is its retrospective observational method and absence of a control group without fatty liver disease. Biopsy is known as the gold standard but invasive procedure for NAFLD diagnosis, thus we limited our analysis to the ultrasound results in the patients’ file (1, 51). The third limitation of this study may be attributed to the adjustments. Despite all of the adjustments done in the analyses, it is still possible that unmeasured confounders such as the current use of chronic medications (glucose lowering, antiplatelet agents, lipid lowering and cardioprotective agents), occupation, daily activity, physical activity and dietary habits can potentially interfere with part of the associations. Another remarkable limitation of our study is failure to follow the patients assessing the risk of developing CVD events or diabetes mellitus within time. Although this issue significantly questions the design of our study, further investigations are strongly recommended.

Therefore, we can claim that this population can be a sample of almost the entire fatty liver community in this region. Also, very few studies on the frequency of pre-diabetes have been conducted in the NAFLD population. All in all, the main scope of conducting such studies is to detect the cases prone to disease related to insulin resistance and metabolic syndrome in early stages and provide a thorough guideline to prevent from the progression of the condition toward life-threatening CVDs and reduce the burden of treatment. We have previously investigated this association in the population of diabetic individuals (8); nevertheless, preventive strategies in prediabetes might have significantly positive effects on the health of general population, mostly those who are not aware of their threatened health such as the individuals with NAFLD and prediabetes. Given that, further larger screening schedules in the community of Iran are recommended to explore the individuals with NAFLD and prediabetes or diabetes mellitus and initiate medications by which hepato- and cardioprotection can be achieved. Although we have not evaluated the impact of medications in preventive setting on NAFLD, prediabetes and CVDs, evidence in the literature have presented favorable outcomes. However, rarely studies have focused on prediabetes and the major body of evidence have concentrated on NAFLD and diabetes (52, 53). Drugs such as the insulin-sensitizing thiazolidinediones, glucagon-like peptide-1 (GLP-1) agonists and sodium-glucose Cotransporter-2 (SGLT2) Inhibitors have prompted promising outcomes in this regard (53–57).

## Conclusion

In our study, nearly one-third of the population of NAFLD patients are pre-diabetic. We found a significant relationship between age, BMI, abnormal WHR, TG, and metabolic syndrome with pre-diabetes compared to normal glucose NAFLD cases. Moreover, pre-diabetes in NAFLD was a predicting factor for metabolic syndrome, but not associated with increased risk of CVD. In order to improve the generalizability of the association between cardiovascular disease and NAFLD, it is recommended to conduct further studies with prospective design in different regions and various subpopulations.

## Data Availability

The raw data supporting the conclusions of this article will be made available by the authors, without undue reservation.
